# Supplementary figures and images for: Quantitative mechanistic model reveals key determinants of placental IgG transfer and informs prenatal immunization strategies
Source: PLoS Comput Biol. 2023 Nov 7;19(11):e1011109. doi: 10.1371/journal.pcbi.1011109 (PMC10656024; doi:10.1371/journal.pcbi.1011109)

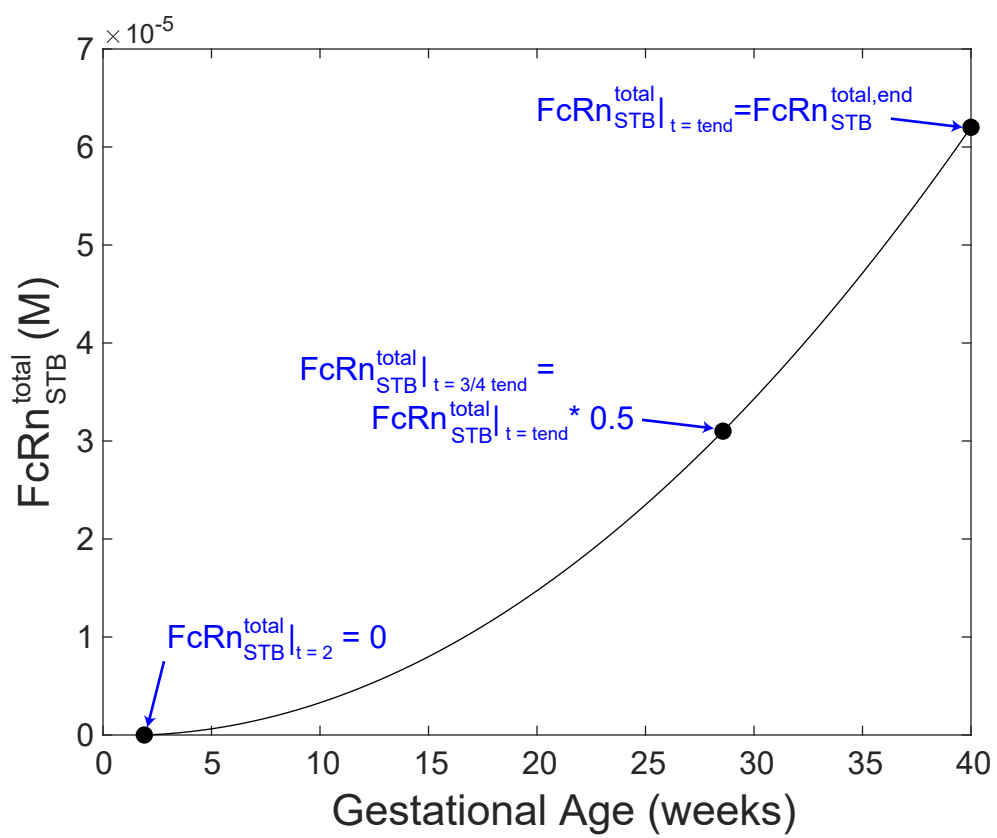

Supplement: S1 Fig — FcRnSTB, FcRnEC, and FcγRIIbEC were modeled as second order polynomials based on trends observed in rat placenta in a previous study by Wang et al [31]. The fit for FcRnSTB is shown here as an example. The trend of FcRnSTB protein expression levels in rat placenta was scaled to the length of human gestation. The concentration of FcRnSTB at full term was an optimized parameter. Additional constraints to achieve similar convex dynamics as observed by Wang et al. [31] are shown in blue. See Equation 13 (S1 Appendix) for more details. (PDF) [file pcbi.1011109.s001.pdf]

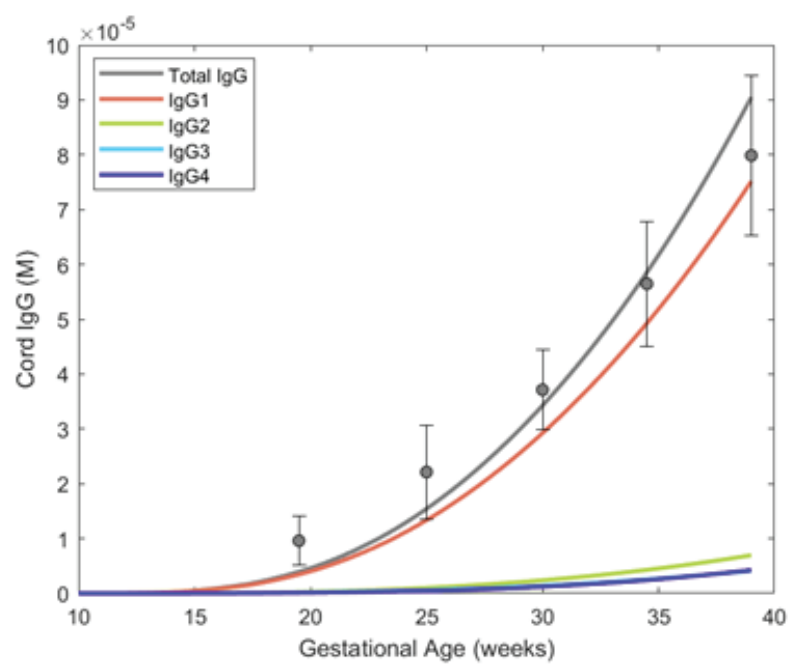

Supplement: S2 Fig — Bulk IgG in umbilical cord blood measured using cordocentesis in a cohort spanning gestational ages is shown as the cohort mean and standard deviation in grey circles. Simulated total fetal IgG and IgG subclasses across gestation are overlaid as solid curve. The curves for IgG3 and IgG4 are overlapping. (PDF) [file pcbi.1011109.s002.pdf]

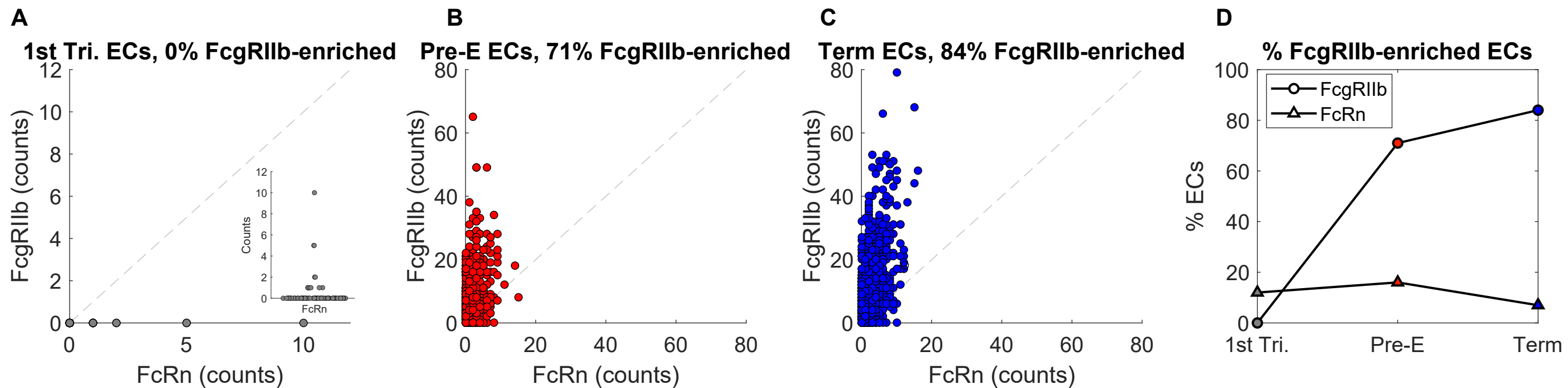

Supplement: S3 Fig — (A-C) The scatter plots show the expression of FCGR2B and FCGRT in placental ECs in scRNA-seq data sets from three gestational time points: (A) first trimester, (B) early third trimester, and (C) late third trimester. Each dot corresponds to a single cell. Line y = x is indicated on each panel. In panel (A), a swarmchart of FcRn expression is included as an inlet to demonstrate the abundance of cells more clearly at the origin. (D) The trend of FcRn and FcγRIIb expression is shown across gestation as the percentage of ECs expressing FcγRIIb (circles) and FcRn (triangles) at each time point. (PDF) [file pcbi.1011109.s003.pdf]

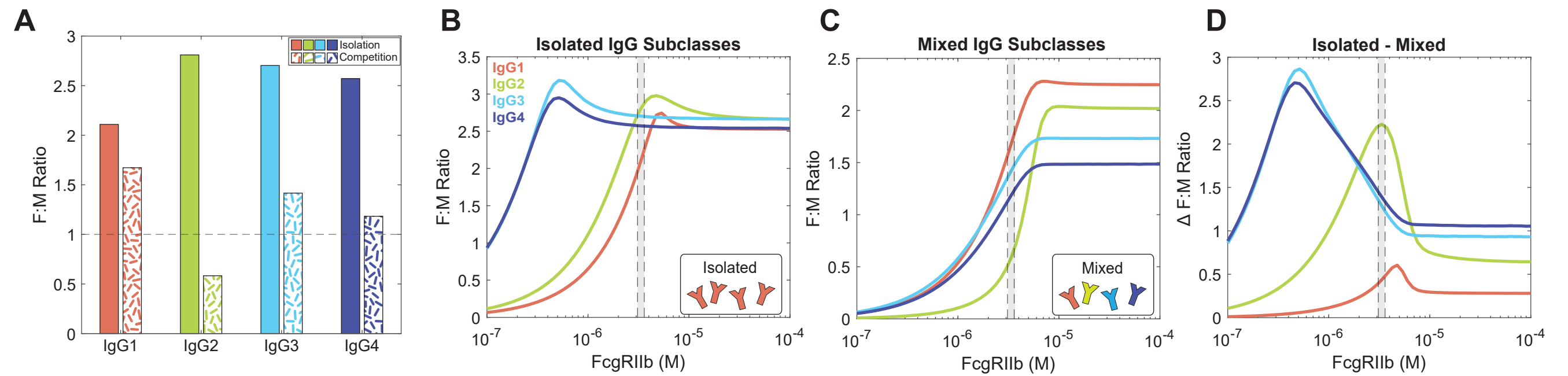

Supplement: S4 Fig — (A) The bar plot represents the F:M ratio at parturition for a term pregnancy from independent simulations in the compartmental placental model with each subclass isolated (solid fill) or mixed with other IgG subclasses (“Competition”, patterned fill). (B-C) The F:M ratio at parturition for a term pregnancy is shown for each subclass in isolation (B) or mixed with other subclasses (C) as in (A) over a range of FcγRIIb expression. (D) The difference (delta) in F:M ratio at parturition for a term pregnancy between isolation and competition conditions is shown over a range of FcγRIIb expression. In (B-D), the optimized range of FcγRIIb expression is indicated by the shaded region. (PDF) [file pcbi.1011109.s004.pdf]

**A**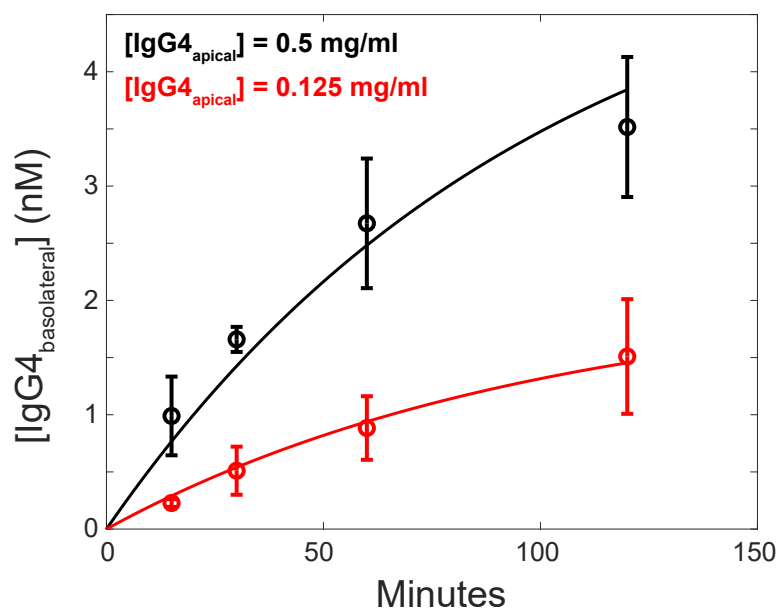**B**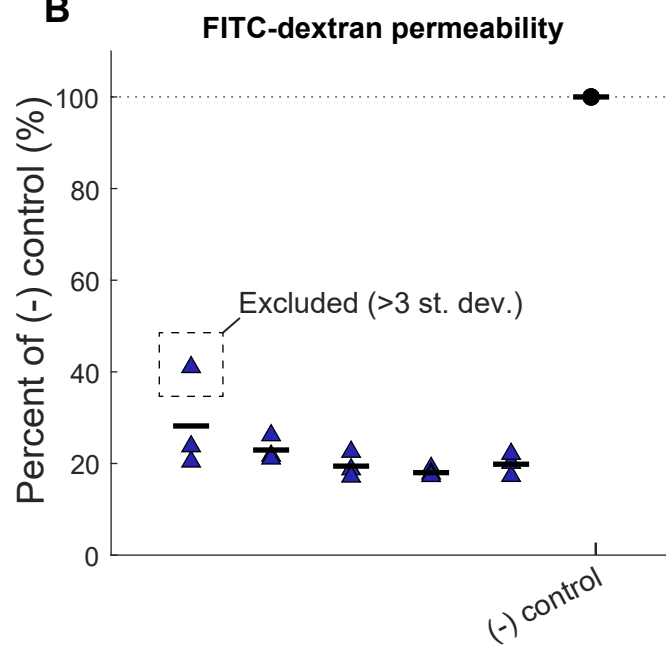

Supplement: S5 Fig — (A) Longitudinal dynamic data from IgG transcytosis measured in vitro in a HUVEC model are shown as the mean and standard deviation of 3 replicates. The overlaid curves show corresponding model simulations described in Methods. (B) The FITC-dextran permeability measurements demonstrates the criteria for rejecting a data point on the basis of faulty monolayer formation. (PDF) [file pcbi.1011109.s005.pdf]

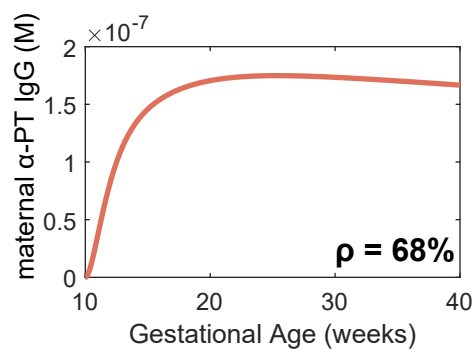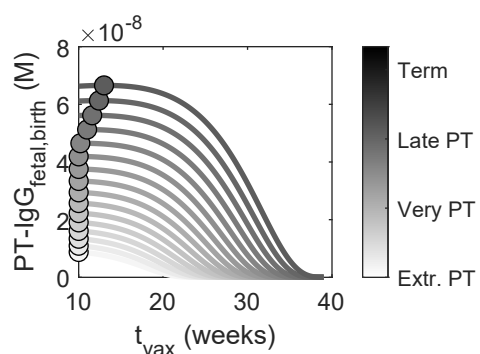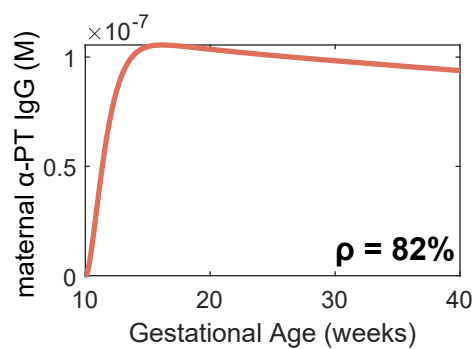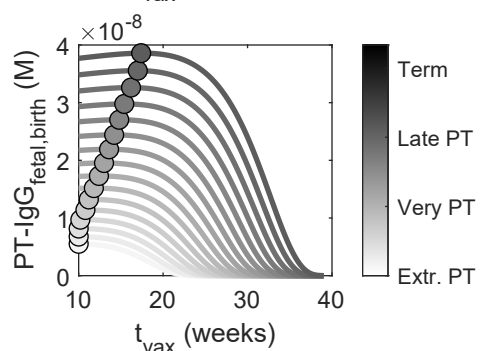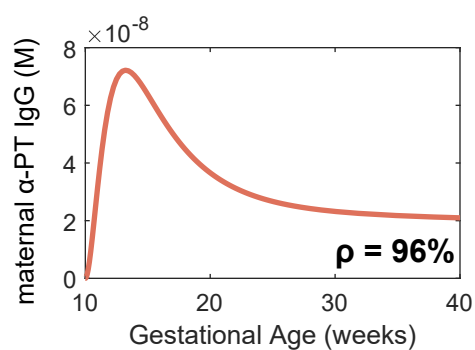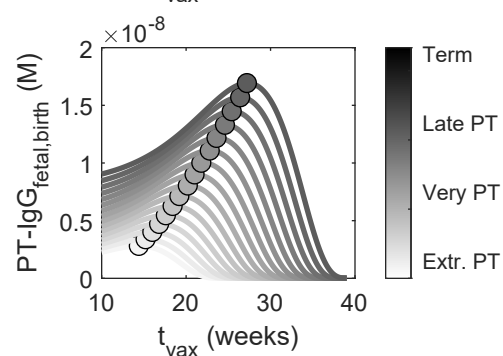

Supplement: S6 Fig — The existence of a transient spike in α-PT IgG is driven by parameter ρ, the proportion of short-lived antibody secreting cells. Three cases are shown within the range of optimized values of ρ reported in White et al.[45] When ρ = 68% (top), the antibody response is driven by a larger proportion of long-lived antibody secreting cells resulting in a persistently high level of anti-PT IgG. Consequently, optimal immunization times were earlier in gestation for the majority of gestational age groups. When ρ = 82% (middle), the antibody response peaks and gradually declines and optimal immunization times were between 10–19 weeks gestation. When ρ = 96% (bottom), the spike in maternal IgG levels is highly transient and optimal immunization time scales with gestational age. (PDF) [file pcbi.1011109.s006.pdf]

**A**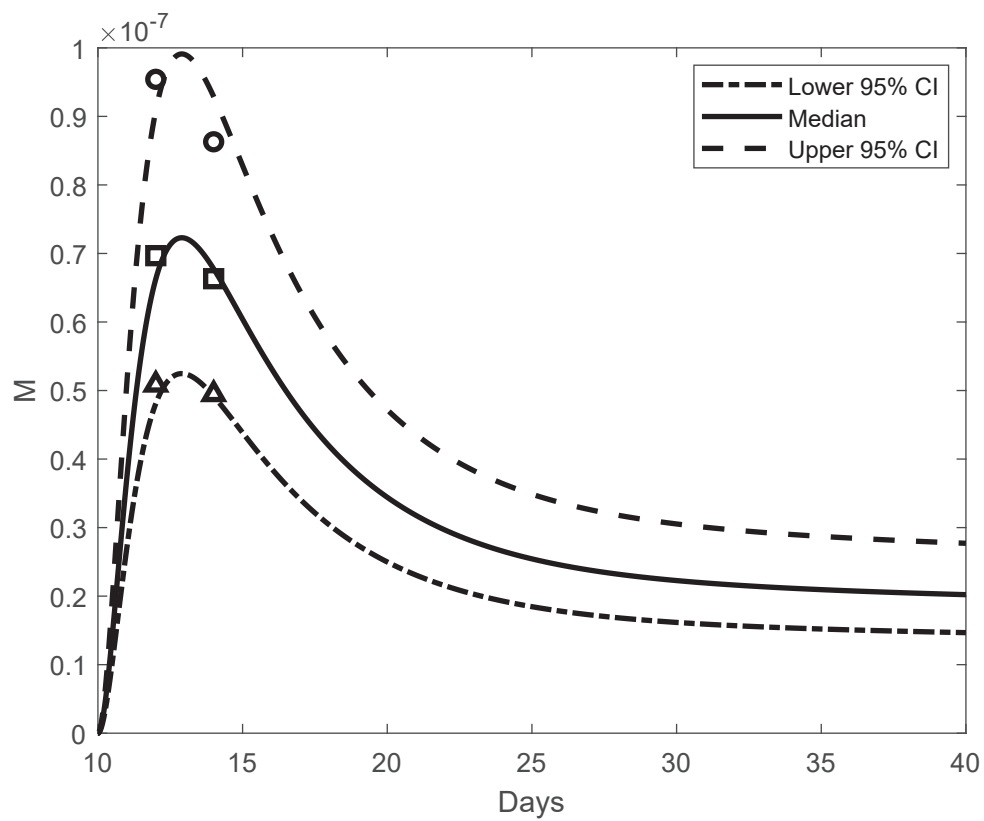**B**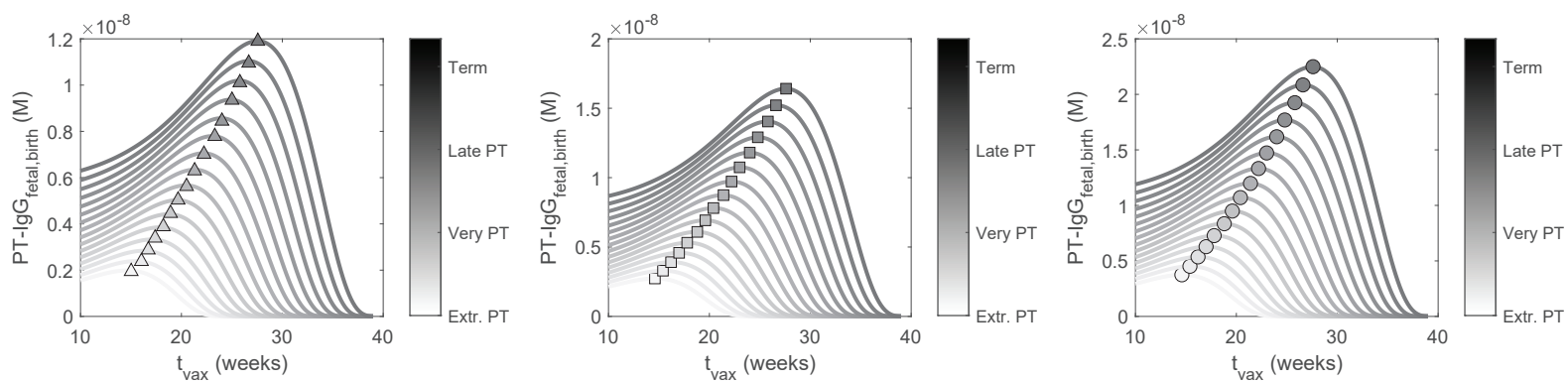

Supplement: S7 Fig — Three models of the maternal α-PT IgG response to Tdap immunization were fit to the median (squares), upper (circles), and lower (triangles) 95% confidence intervals reported by van der Lee et al. [47] The magnitude of the maternal α-PT IgG spike affected the level of α-PT IgG present in the fetus at the time of delivery but did not impact timing of the optimal immunization window. (PDF) [file pcbi.1011109.s007.pdf]
