# Supplementary material for: Quantitative mechanistic model reveals key determinants of placental IgG transfer and informs prenatal immunization strategies
Source: PLoS Comput Biol. 2023 Nov 7;19(11):e1011109. doi: 10.1371/journal.pcbi.1011109 (PMC10656024; doi:10.1371/journal.pcbi.1011109)
Supplement: S1 Appendix — (PDF) [file pcbi.1011109.s008.pdf]

S1 Appendix. Placental IgG transfer model equations.

Equations 1-4 represent IgG concentrations in the maternal plasma compartment:

$$\frac{dIgG1_M}{dt} = IgG1_M(k_{gen} - k_{up}) = 0 \quad (1)$$

$$\frac{dIgG2_M}{dt} = IgG2_M(k_{gen} - k_{up}) = 0 \quad (2)$$

$$\frac{dIgG3_M}{dt} = IgG3_M(k_{gen} - k_{up}) = 0 \quad (3)$$

$$\frac{dIgG4_M}{dt} = IgG4_M(k_{gen} - k_{up}) = 0 \quad (4)$$

Given the simplifying assumption that the overall concentration of IgG in the mother is constant such that the rate of new antibody generation (k_{gen}) and uptake by placental STBs (k_{up}) are equal. Equations 5-12 represent IgG in STB endosomes, either as monovalent IgG (5-8) or IgG in complex with FcRn in STB endosomes (9-12):

$$\frac{dIgG1_{STB}}{dt} = [k_{off}^{IgG1,FcRn} C_{STB}^{IgG1,FcRn} + k_{up} IgG1_M - k_{on}^{IgG1,FcRn} IgG1_{STB} FcRn_{STB}^{free} - k_{deg} IgG1_{STB}] \frac{1}{v_{STB}} \quad (5)$$

$$\frac{dIgG2_{STB}}{dt} = [k_{off}^{IgG2,FcRn} C_{STB}^{IgG2,FcRn} + k_{up} IgG2_M - k_{on}^{IgG2,FcRn} IgG2_{STB} FcRn_{STB}^{free} - k_{deg} IgG2_{STB}] \frac{1}{v_{STB}} \quad (6)$$

$$\frac{dIgG3_{STB}}{dt} = [k_{off}^{IgG3,FcRn} C_{STB}^{IgG3,FcRn} + k_{up} IgG3_M - k_{on}^{IgG3,FcRn} IgG3_{STB} FcRn_{STB}^{free} - k_{deg} IgG3_{STB}] \frac{1}{v_{STB}} \quad (7)$$

$$\frac{dIgG4_{STB}}{dt} = [k_{off}^{IgG4,FcRn} C_{STB}^{IgG4,FcRn} + k_{up} IgG4_M - k_{on}^{IgG4,FcRn} IgG4_{STB} FcRn_{STB}^{free} - k_{deg} IgG4_{STB}] \frac{1}{v_{STB}} \quad (8)$$

$$\frac{dC_{STB}^{IgG1,FcRn}}{dt} = [-k_{off}^{IgG1,FcRn}C_{STB}^{IgG1,FcRn} + k_{on}^{IgG1,FcRn}IgG1_{STB}FcRn_{STB}^{free} - k_{trans}C_{STB}^{IgG1,FcRn}]\frac{1}{v_{STB}} \quad (9)$$

$$\frac{dC_{STB}^{IgG2,FcRn}}{dt} = \left[-k_{off}^{IgG2,FcRn}C_{STB}^{IgG2,FcRn} + k_{on}^{IgG2,FcRn}IgG2_{STB}FcRn_{STB}^{free} - k_{trans}C_{STB}^{IgG2,FcRn}\right]\frac{1}{v_{STB}} \quad (10)$$

$$\frac{dC_{STB}^{IgG3,FcRn}}{dt} = \left[-k_{off}^{IgG3,FcRn}C_{STB}^{IgG3,FcRn} + k_{on}^{IgG3,FcRn}IgG3_{STB}FcRn_{STB}^{free} - k_{trans}C_{STB}^{IgG3,FcRn}\right]\frac{1}{v_{STB}} \quad (11)$$

$$\frac{dC_{STB}^{IgG4,FcRn}}{dt} = \left[-k_{off}^{IgG4,FcRn}C_{STB}^{IgG4,FcRn} + k_{on}^{IgG4,FcRn}IgG4_{STB}FcRn_{STB}^{free} - k_{trans}C_{STB}^{IgG4,FcRn}\right]\frac{1}{v_{STB}} \quad (12)$$

Equation 13 represents the concentration of free, unbound FcRn expressed within STB endosomes:

$$\begin{aligned}\frac{dFcRn_{STB}^{free}}{dt} = [&k_{off}^{IgG1,FcRn}C_{STB}^{IgG1,FcRn} + k_{off}^{IgG2,FcRn}C_{STB}^{IgG2,FcRn} + k_{off}^{IgG3,FcRn}C_{STB}^{IgG3,FcRn} \\ &+ k_{off}^{IgG4,FcRn}C_{STB}^{IgG4,FcRn} \\ &- \left(k_{on}^{IgG1,FcRn}IgG1_{STB} + k_{on}^{IgG2,FcRn}IgG2_{STB} + k_{on}^{IgG3,FcRn}IgG3_{STB} \right. \\ &\left. + k_{on}^{IgG4,FcRn}IgG4_{STB}\right)FcRn_{STB}^{free} \\ &+ k_{trans}\left(C_{STB}^{IgG1,FcRn} + C_{STB}^{IgG2,FcRn} + C_{STB}^{IgG3,FcRn} + C_{STB}^{IgG4,FcRn}\right) \\ &+ \Delta FcRn_{STB}^{total}]\frac{1}{v_{STB}} \quad (13)\end{aligned}$$

Where the total concentration of FcRn expressed by STBs increases exponentially across gestation, and the derivative of the second order polynomial curve fit ($\Delta FcRn_{STB}^{total}$) represents the increase in total FcRn at each time step:

$$FcRn_{STB}^{total} = at^2 + bt + c = FcRn_{STB}^{free} + [FcRn - \sum_i C_{STB}^{IgGi,FcRn}] \quad (13.i)$$

$$\Delta FcRn_{STB}^{total} = 2at + b \quad (13.ii)$$

Where parameters a, b, and c determined given the following conditions:

$$FcRn_{STB}^{total}|_{t=10} = 0$$

$$FcRn_{STB}^{total}|_{t=\frac{3}{4}t_{end}} = \frac{FcRn_{STB}^{total,end}}{2}$$

$$FcRn_{STB}^{total}|_{t=t_{end}} = FcRn_{STB}^{total,end}$$

And $FcRn_{STB}^{total,end}$ is a parameter optimized by CaliPro (Table 1). These assumptions are based on FcRn expression data in rat placenta scaled to the length of human gestation reported in Wang *et al* (31).

FcRn binding results in IgG transcytosis through STBs and into the intervillous stroma. IgG concentration in the stroma is represented by equations 14-17:

$$\frac{dIgG1_{STR}}{dt} = [k_{trans}C_{STB}^{IgG1,FcRn} + k_{off}^{IgG1,Fc\gamma RIIb}C_{EC}^{IgG1,Fc\gamma RIIb} - k_{on}^{IgG1,Fc\gamma RIIb}IgG1_{STR}Fc\gamma RIIb_{EC}^{free} - k_{up}IgG1_{STR}]\frac{1}{v_{STR}} \quad (14)$$

$$\frac{dIgG2_{STR}}{dt} = [k_{trans}C_{STB}^{IgG2,FcRn} + k_{off}^{IgG2,Fc\gamma RIIb}C_{EC}^{IgG2,Fc\gamma RIIb} - k_{on}^{IgG2,Fc\gamma RIIb}IgG2_{STR}Fc\gamma RIIb_{EC}^{free} - k_{up}IgG2_{STR}]\frac{1}{v_{STR}} \quad (15)$$

$$\frac{dIgG3_{STR}}{dt} = [k_{trans}C_{STB}^{IgG3,FcRn} + k_{off}^{IgG3,Fc\gamma RIIb}C_{EC}^{IgG3,Fc\gamma RIIb} - k_{on}^{IgG3,Fc\gamma RIIb}IgG3_{STR}Fc\gamma RIIb_{EC}^{free} - k_{up}IgG3_{STR}]\frac{1}{v_{STR}} \quad (16)$$

$$\frac{dIgG4_{STR}}{dt} = [k_{trans}C_{STB}^{IgG4,FcRn} + k_{off}^{IgG4,Fc\gamma RIIb}C_{EC}^{IgG4,Fc\gamma RIIb} - k_{on}^{IgG4,Fc\gamma RIIb}IgG4_{STR}Fc\gamma RIIb_{EC}^{free} - k_{up}IgG4_{STR}]\frac{1}{v_{STR}} \quad (17)$$

Stromal IgG binds to FcγRIIb on the EC surface. The concentration of IgG subclasses and FcγRIIb in complex formation on the EC surface is given by equations 18-21:

$$\frac{dC_{EC}^{IgG1,Fc\gamma RIIb}}{dt} = \left[-k_{off}^{IgG1,Fc\gamma RIIb}C_{EC}^{IgG1,Fc\gamma RIIb} + k_{on}^{IgG1,Fc\gamma RIIb}IgG1_{STR}Fc\gamma RIIb_{EC}^{free} - k_{trans}C_{EC}^{IgG1,Fc\gamma RIIb}\right]\frac{1}{v_{STR}} \quad (18)$$

$$\frac{dC_{EC}^{IgG2,Fc\gamma RIIb}}{dt} = \Big[-k_{off}^{IgG2,Fc\gamma RIIb} C_{EC}^{IgG2,Fc\gamma RIIb} + k_{on}^{IgG2,Fc\gamma RIIb} IgG2_{STR} Fc\gamma RIIb_{EC}^{free} - k_{trans} C_{EC}^{IgG2,Fc\gamma RIIb}\Big] \frac{1}{v_{STR}} \quad (19)$$

$$\frac{dC_{EC}^{IgG3,Fc\gamma RIIb}}{dt} = \Big[-k_{off}^{IgG3,Fc\gamma RIIb} C_{EC}^{IgG3,Fc\gamma RIIb} + k_{on}^{IgG3,Fc\gamma RIIb} IgG3_{STR} Fc\gamma RIIb_{EC}^{free} - k_{trans} C_{EC}^{IgG3,Fc\gamma RIIb}\Big] \frac{1}{v_{STR}} \quad (20)$$

$$\frac{dC_{EC}^{IgG4,Fc\gamma RIIb}}{dt} = \Big[-k_{off}^{IgG4,Fc\gamma RIIb} C_{EC}^{IgG4,Fc\gamma RIIb} + k_{on}^{IgG4,Fc\gamma RIIb} IgG4_{STR} Fc\gamma RIIb_{EC}^{free} - k_{trans} C_{EC}^{IgG4,Fc\gamma RIIb}\Big] \frac{1}{v_{STR}} \quad (21)$$

And the concentration of free FcγRIIb is given by equation 22:

$$\frac{dFc\gamma RIIb_{EC}^{free}}{dt} = [k_{off}^{IgG1,Fc\gamma RIIb} C_{EC}^{IgG1,Fc\gamma RIIb} + k_{off}^{IgG2,Fc\gamma RIIb} C_{EC}^{IgG2,Fc\gamma RIIb} + k_{off}^{IgG3,Fc\gamma RIIb} C_{EC}^{IgG3,Fc\gamma RIIb} + k_{off}^{IgG4,Fc\gamma RIIb} C_{EC}^{IgG4,Fc\gamma RIIb} - \big(k_{on}^{IgG1,Fc\gamma RIIb} IgG1_{STR} + k_{on}^{IgG2,Fc\gamma RIIb} IgG2_{STR} + k_{on}^{IgG3,Fc\gamma RIIb} IgG3_{STR} + k_{on}^{IgG4,Fc\gamma RIIb} IgG4_{STR}\big) Fc\gamma RIIb_{EC}^{free} + k_{trans}\big(C_{EC}^{IgG1,Fc\gamma RIIb} + C_{EC}^{IgG2,Fc\gamma RIIb} + C_{EC}^{IgG3,Fc\gamma RIIb} + C_{EC}^{IgG4,Fc\gamma RIIb}\big) + \Delta Fc\gamma RIIb_{EC}^{total}] \frac{1}{v_{STR}} \quad (22)$$

Where $\Delta Fc\gamma RIIb_{EC}^{total}$ is the derivative of the second order polynomial curve fit representing increasing FcγRIIb concentration across gestation:

$$Fc\gamma RIIb_{EC}^{total} = at^2 + bt + c \quad (22.i)$$

$$\Delta Fc\gamma RIIb_{EC}^{total} = 2at + b \quad (22.ii)$$

Where a, b, and c are parameters determined given a value FcγRIIb at term ($Fc\gamma RIIb_{EC}^{total,end}$) and similar constraints as described in equations (13.i – 13.ii). FcγRIIb binding results in IgG

being taken up into EC endosomes where IgG dissociates from FcγRIIb and is trafficked across the ECs.

In contrast, EC FcRn binds IgG at an acidic pH within EC endosomes, which is modeled as:

$$\frac{dFcRn_{EC}^{free}}{dt} = [k_{off}^{IgG1,FcRn}C_{EC}^{IgG1,FcRn} + k_{off}^{IgG2,FcRn}C_{EC}^{IgG2,FcRn} + k_{off}^{IgG3,FcRn}C_{EC}^{IgG3,FcRn} + k_{off}^{IgG4,FcRn}C_{EC}^{IgG4,FcRn} - \left(k_{on}^{IgG1,FcRn}IgG1_{EC} + k_{on}^{IgG2,FcRn}IgG2_{EC} + k_{on}^{IgG3,FcRn}IgG3_{EC} + k_{on}^{IgG4,FcRn}IgG4_{EC}\right)FcRn_{EC}^{free} + k_{trans}\left(C_{EC}^{IgG1,FcRn} + C_{EC}^{IgG2,FcRn} + C_{EC}^{IgG3,FcRn} + C_{EC}^{IgG4,FcRn}\right) + \Delta FcRn_{EC}^{total}]\frac{1}{v_{EC}} \quad (23)$$

Where $\Delta FcRn_{EC}^{total}$ is the derivative of the second order polynomial curve fit representing increasing FcRn concentration across gestation (S1 Fig):

$$FcRn_{EC}^{total} = at^2 + bt + c \quad (23.i)$$

$$\Delta FcRn_{EC}^{total} = 2at + b \quad (23.ii)$$

Where a, b, and c are parameters determined given a value FcRn at term ($FcRn_{EC}^{total,end}$) and similar constraints as described in equations (13.i – 13.ii). The concentration of FcRn-IgG complexes within EC endosomes is given by equations 24-27:

$$\frac{dC_{EC}^{IgG1,FcRn}}{dt} = \left[-k_{off}^{IgG1,FcRn}C_{EC}^{IgG1,FcRn} + k_{on}^{IgG1,FcRn}IgG1_{EC}FcRn_{EC}^{free} - k_{trans}C_{EC}^{IgG1,FcRn}\right]\frac{1}{v_{EC}} \quad (24)$$

$$\frac{dC_{EC}^{IgG2,FcRn}}{dt} = \left[-k_{off}^{IgG2,FcRn}C_{EC}^{IgG2,FcRn} + k_{on}^{IgG2,FcRn}IgG2_{EC}FcRn_{EC}^{free} - k_{trans}C_{EC}^{IgG2,FcRn}\right]\frac{1}{v_{EC}} \quad (25)$$

$$\frac{dC_{EC}^{IgG3,FcRn}}{dt} = \left[-k_{off}^{IgG3,FcRn}C_{EC}^{IgG3,FcRn} + k_{on}^{IgG3,FcRn}IgG3_{EC}FcRn_{EC}^{free} - k_{trans}C_{EC}^{IgG3,FcRn}\right]\frac{1}{v_{EC}} \quad (26)$$

$$\frac{dC_{EC}^{IgG4,FcRn}}{dt} = \left[-k_{off}^{IgG4,FcRn}C_{EC}^{IgG4,FcRn} + k_{on}^{IgG4,FcRn}IgG4_{EC}FcRn_{EC}^{free} - k_{trans}C_{EC}^{IgG4,FcRn}\right]\frac{1}{v_{EC}} \quad (27)$$

The concentration of free IgG within EC endosomes is given by equations 28-32:

$$\frac{dIgG1_{EC}}{dt} = [k_{up}IgG1_{STR} - k_{on}^{IgG1,FcRn}IgG1_{EC}FcRn_{EC}^{free} + k_{off}^{IgG1,FcRn}C_{EC}^{IgG1,FcRn} - k_{deg}IgG1_{EC}]\frac{1}{v_{EC}} \quad (28)$$

$$\frac{dIgG2_{EC}}{dt} = [k_{up}IgG2_{STR} - k_{on}^{IgG2,FcRn}IgG2_{EC}FcRn_{EC}^{free} + k_{off}^{IgG2,FcRn}C_{EC}^{IgG2,FcRn} - k_{deg}IgG2_{EC}]\frac{1}{v_{EC}} \quad (29)$$

$$\frac{dIgG3_{EC}}{dt} = \left[k_{up}IgG3_{STR} - k_{on}^{IgG3,FcRn}IgG3_{EC}FcRn_{EC}^{free} + k_{off}^{IgG3,FcRn}C_{EC}^{IgG3,FcRn} - k_{deg}IgG3_{EC}\right]\frac{1}{v_{EC}} \quad (30)$$

$$\frac{dIgG4_{EC}}{dt} = \left[k_{up}IgG4_{STR} - k_{on}^{IgG4,FcRn}IgG4_{EC}FcRn_{EC}^{free} + k_{off}^{IgG4,FcRn}C_{EC}^{IgG4,FcRn} - k_{deg}IgG4_{EC}\right]\frac{1}{v_{EC}} \quad (31)$$

The IgG concentration in the fetus is represented by equations 32-35:

$$\frac{dIgG1_{F}}{dt} = [k_{trans}(C_{EC}^{IgG1,FcRn} + C_{EC}^{IgG1,Fc\gamma RIIb}) - \delta_{Ab}]\frac{1}{v_{F}} \quad (32)$$

$$\frac{dIgG2_{F}}{dt} = \left[k_{trans}(C_{EC}^{IgG2,FcRn} + C_{EC}^{IgG2,Fc\gamma RIIb}) - \delta_{Ab}\right]\frac{1}{v_{F}} \quad (33)$$

$$\frac{dIgG3_{F}}{dt} = \left[k_{trans}(C_{EC}^{IgG3,FcRn} + C_{EC}^{IgG3,Fc\gamma RIIb}) - \delta_{Ab}\right]\frac{1}{v_{F}} \quad (34)$$

$$\frac{dIgG4_{F}}{dt} = [k_{trans}(C_{EC}^{IgG4,FcRn} + C_{EC}^{IgG4,Fc\gamma RIIb}) - \delta_{Ab}]\frac{1}{v_{F}} \quad (35)$$
