# Supplementary material for: Quantitative mechanistic model reveals key determinants of placental IgG transfer and informs prenatal immunization strategies
Source: PLoS Comput Biol. 2023 Nov 7;19(11):e1011109. doi: 10.1371/journal.pcbi.1011109 (PMC10656024; doi:10.1371/journal.pcbi.1011109)
Supplement: S2 Appendix — (PDF) [file pcbi.1011109.s009.pdf]

S2 Appendix. Tdap immunization model equations.

Equations 36-39 represent the naïve B cell response to maternal immunization against pertussis toxin. The concentration of antigen injected intramuscularly in the mother is given in equation 36:

$$\frac{dAg}{dt} = \begin{cases} t < t_{vax}, & 0 \\ t \geq t_{vax}, & [-\delta_{Ag} Ag] \dfrac{1}{v_M} \end{cases} \quad (36)$$

And is equal to 0 prior to t_{vax}. Equation 37 and 38 represent the concentration of short-lived antibody secreting cells (S_{ASC}) and long-lived antibody secreting cells (L_{ASC}) specific to pertussis toxin following immunization:

$$\frac{dS_{ASC}}{dt} = \begin{cases} t < t_{vax}, & 0 \\ t \geq t_{vax}, & [\rho k_{ASC} - \delta_{S-ASC} S_{ASC}] \dfrac{1}{v_M} \end{cases} \quad (37)$$

$$\frac{dL_{ASC}}{dt} = \begin{cases} t < t_{vax}, & 0 \\ t \geq t_{vax}, & [(1-\rho) k_{ASC} - \delta_{L-ASC} L_{ASC}] \dfrac{1}{v_M} \end{cases} \quad (38)$$

The concentration of IgG in the mother specific to pertussis toxin is represented by equation 39:

$$\frac{dIgG_M^{\alpha-PT}}{dt} = \begin{cases} t < t_{vax}, & 0 \\ t \geq t_{vax}, & \left[k_{IgG}(S_{ASC} + L_{ASC}) - \delta_{IgG} IgG_M^{\alpha-PT}\right] \dfrac{1}{v_M} \end{cases} \quad (39)$$

To perform pertussis toxin immunization simulations, equations 36-39 are layered into the model described by equations 1-35. New equations similar to 5-12, 14-21, and 24-35 are added to this set to represent anti-pertussis toxin IgG1, IgG2, IgG3, and IgG4 in STB endosomes, the stroma, EC endosomes, and the fetus. Rate-limiting equations 13, 22, and 23 are adjusted to account for competition between bulk IgG1-4 and anti-pertussis toxin IgG1-4 for $FcRn_{STB}$, $Fc\gamma RIIb_{EC}$, and $FcRn_{EC}$ binding for a total of 72 equations:

$$\frac{dFcRn_{STB}^{free}}{dt} = [k_{off}^{IgG1,FcRn}(C_{STB}^{IgG1,FcRn} + C_{STB,\alpha-PT}^{IgG1,FcRn}) + k_{off}^{IgG2,FcRn}(C_{STB}^{IgG2,FcRn} + C_{STB,\alpha-PT}^{IgG2,FcRn})$$
$$+ k_{off}^{IgG3,FcRn}(C_{STB}^{IgG3,FcRn} + +C_{STB,\alpha-PT}^{IgG3,FcRn}) + k_{off}^{IgG4,FcRn}(C_{STB}^{IgG4,FcRn} + +C_{STB,\alpha-PT}^{IgG4,FcRn})$$
$$- \big(k_{on}^{IgG1,FcRn}(IgG1_{STB} + IgG1_{STB}^{\alpha-PT}) + k_{on}^{IgG2,FcRn}(IgG2_{STB} + IgG2_{STB}^{\alpha-PT})$$
$$+ k_{on}^{IgG3,FcRn}(IgG3_{STB} + IgG3_{STB}^{\alpha-PT}) + k_{on}^{IgG4,FcRn}(IgG4_{STB} + IgG4_{STB}^{\alpha-PT})\big)FcRn_{STB}^{free}$$
$$+ k_{trans}\big(C_{STB}^{IgG1,FcRn} + C_{STB}^{IgG2,FcRn} + C_{STB}^{IgG3,FcRn} + C_{STB}^{IgG4,FcRn} + C_{STB,\alpha-PT}^{IgG1,FcRn}$$
$$+ C_{STB,\alpha-PT}^{IgG2,FcRn} + C_{STB,\alpha-PT}^{IgG3,FcRn} + C_{STB,\alpha-PT}^{IgG4,FcRn}\big) + \Delta FcRn_{STB}^{total}]\frac{1}{v_{STB}} \quad (40)$$

$$\frac{dFc\gamma RIIb_{STB}^{free}}{dt} = [k_{off}^{IgG1,Fc\gamma RIIb}(C_{EC}^{IgG1,Fc\gamma RIIb} + C_{EC,\alpha-PT}^{IgG1,Fc\gamma RIIb}) + k_{off}^{IgG2,Fc\gamma RIIb}(C_{EC}^{IgG2,Fc\gamma RIIb}$$
$$+ C_{EC,\alpha-PT}^{IgG2,Fc\gamma RIIb}) + k_{off}^{IgG3,Fc\gamma RIIb}(C_{EC}^{IgG3,Fc\gamma RIIb} + +C_{EC,\alpha-PT}^{IgG3,Fc\gamma RIIb})$$
$$+ k_{off}^{IgG4,Fc\gamma RIIb}(C_{EC}^{IgG4,Fc\gamma RIIb} + +C_{EC,\alpha-PT}^{IgG4,Fc\gamma RIIb})$$
$$- \big(k_{on}^{IgG1,Fc\gamma RIIb}(IgG1_{STR} + IgG1_{STR}^{\alpha-PT}) + k_{on}^{IgG2,Fc\gamma RIIb}(IgG2_{STR} + IgG2_{STR}^{\alpha-PT})$$
$$+ k_{on}^{IgG3,Fc\gamma RIIb}(IgG3_{STR} + IgG3_{STR}^{\alpha-PT}) + k_{on}^{IgG4,Fc\gamma RIIb}(IgG4_{STR}$$
$$+ IgG4_{STR}^{\alpha-PT})\big)Fc\gamma RIIb_{EC}^{free}$$
$$+ k_{trans}\big(C_{EC}^{IgG1,Fc\gamma RIIb} + C_{EC}^{IgG2,Fc\gamma RIIb} + C_{EC}^{IgG3,Fc\gamma RIIb} + C_{EC}^{IgG4,Fc\gamma RIIb} + C_{EC,\alpha-PT}^{IgG1,Fc\gamma RIIb}$$
$$+ C_{EC,\alpha-PT}^{IgG2,Fc\gamma RIIb} + C_{EC,\alpha-PT}^{IgG3,Fc\gamma RIIb} + C_{EC,\alpha-PT}^{IgG4,Fc\gamma RIIb}\big) + \Delta Fc\gamma RIIb_{EC}^{total}]\frac{1}{v_{EC}} \quad (41)$$

$$\frac{dFcRn_{EC}^{free}}{dt} = [k_{off}^{IgG1,FcRn}(C_{EC}^{IgG1,FcRn} + C_{EC,\alpha-PT}^{IgG1,FcRn}) + k_{off}^{IgG2,FcRn}(C_{EC}^{IgG2,FcRn} + C_{EC,\alpha-PT}^{IgG2,FcRn})$$
$$+ k_{off}^{IgG3,FcRn}(C_{EC}^{IgG3,FcRn} + +C_{EC,\alpha-PT}^{IgG3,FcRn}) + k_{off}^{IgG4,FcRn}(C_{EC}^{IgG4,FcRn} + +C_{EC,\alpha-PT}^{IgG4,FcRn})$$
$$- \left(k_{on}^{IgG1,FcRn}(IgG1_{EC} + IgG1_{EC}^{\alpha-PT}) + k_{on}^{IgG2,FcRn}(IgG2_{EC} + IgG2_{EC}^{\alpha-PT})\right.$$
$$\left.+ k_{on}^{IgG3,FcRn}(IgG3_{EC} + IgG3_{EC}^{\alpha-PT}) + k_{on}^{IgG4,FcRn}(IgG4_{EC} + IgG4_{EC}^{\alpha-PT})\right)FcRn_{EC}^{free}$$
$$+ k_{trans}\left(C_{EC}^{IgG1,FcRn} + C_{EC}^{IgG2,FcRn} + C_{EC}^{IgG3,FcRn} + C_{EC}^{IgG4,FcRn} + C_{EC,\alpha-PT}^{IgG1,FcRn}\right.$$
$$\left.+ C_{EC,\alpha-PT}^{IgG2,FcRn} + C_{EC,\alpha-PT}^{IgG3,FcRn} + C_{EC,\alpha-PT}^{IgG4,FcRn}\right) + \Delta FcRn_{EC}^{total}]\frac{1}{v_{EC}} \quad (42)$$
