# Supplementary material for: Quantitative mechanistic model reveals key determinants of placental IgG transfer and informs prenatal immunization strategies
Source: PLoS Comput Biol. 2023 Nov 7;19(11):e1011109. doi: 10.1371/journal.pcbi.1011109 (PMC10656024; doi:10.1371/journal.pcbi.1011109)
Supplement: Attachment — Submitted filename: response to reviewers Erdogan Dolatshahi.pdf [file pcbi.1011109.s011.pdf]

Please find a revised version of our manuscript entitled “*Quantitative mechanistic model reveals key determinants of placental IgG transfer and informs prenatal immunization strategies*” for consideration at the PLOS Computational Biology journal.

We thank the reviewers for their constructive feedback and suggestions. We were pleased that the majority of the critiques were related to the writing clarity, justification of model assumptions, and clarification of model-driven insights and novelty. Briefly, we have carefully revised the manuscript by including the following changes:

- We made edits throughout all sections of the manuscript to address specific comments from reviewers.
- We revised a few model assumptions in response to the reviewers’ suggestions, resulting in changes to Figures 1, 2, 3, 5, and 6, an updated parameter set presented in Table 1, and slightly updated equations in the supplemental information. While these changes did not drastically change the model-driven insights, they improved the model utility for predictive studies by future researchers.
- We made changes to Figures 1, 3, 4, and 6 to address specific feedback from the reviewers and to improve readability of schematic diagrams.
- We added new supplemental Figures S1, S6, and S7 in response to suggestions from both reviewers.
- We added additional references as appropriate to (i) justify model assumptions and (ii) clarify this study’s relationship to prior literature, specifically distinguishing the corroboration of previous observations versus novel insights derived from the model.

Again, we thank the editors and reviewers for this opportunity to improve our manuscript. Our specific responses to each comment from the Reviewers are detailed below.

Reviewer #1

Erdogan and Dolatshahi explored how maternal antibodies are transferred to the fetus in utero using a mechanistic model. Specifically, they constructed a model for FcRn- and FcgRIIb-mediated IgG transport on STBs and ECs and analyzed the sensitivity of each parameter. They also explored the role of FcgRIIb in IgG transfer across fetal ECs as well as evidence of subclass competition. Finally, they applied the model to identify techniques for optimizing vaccination scheduling. Studying placental antibody transfer is highly relevant to maternal health, and this study has the potential to impact vaccine design and timing. However, I have several concerns, about assumptions from the model and questions regarding what kind of actionable insights can be derived from it, that would need to be addressed before publication.

Major issues:

The authors need to clearly define which observations are insights derived from the present study versus prior literature. For example, the propensity for transfer of each subclass is derived from previous studies, and so steering vaccine subclass production, as discussed at length in the discussion, does not seem like an implication of this work. The dynamical nature of the model does seem like a unique contribution that the authors could emphasize more.

We thank the reviewer for this comment and agree that there was some ambiguity in our original draft regarding which observations are derived from this study versus those which corroborate previous findings and serve as model validation. We have carefully revised the manuscript to

make these distinctions and added additional citations as applicable, particularly in the Discussion section. For example, in **lines 467-470** we point towards work by Eberhardt *et al* showing second trimester Tdap vaccination timing may be more efficacious compared to third trimester as evidence that corroborates our model findings (1). Stapleton *et al* revealed that IgG subclasses compete for FcRn binding in trophoblasts, and our study builds upon this work by demonstrating a similar phenomenon is at play with FcgRIIb in ECs, as well as deriving a mathematical formula to quantify the effects of competition (2).

On the other hand, we have revised the text to emphasize novel insights that were only ascertainable using our dynamical modeling approach. For example, our model provides insight into the effects of subclass competition for Fc receptor binding as a mechanism underlying the well-documented phenomenon of subclass-specific IgG transfer. Likewise, in **lines 447-449** we acknowledge work by Ishikawa *et al* and others implicating FcgRIIb in EC transport (3), but we clarify that our study finds FcgRIIb to be a rate-limiting factor contributing to subclass-specific transfer. We further addressed the discrepancy between previous studies regarding whether EC transcytosis is driven by FcRn or FcgRIIb by putting forward that a combination of both FcRn- and FcgRIIb-mediated transport may be implicated in placental ECs (3,4). We revised the Discussion to delineate these unique contributions of our study from prior literature and have done similarly for other points throughout the manuscript.

The practical implications of this model should be clarified. This work pointed out the factors that are positively or negatively associated with earlier or later optimal vaccination time, but many of them are not actionable. E.g., one can’t change their endosomal volume, FcR expression in specific cells, etc. Similarly, this work suggests that the model can be used for personalized medicine. What measurements would be made to calibrate the model for specific individuals?

We thank the reviewer for this comment, because it allowed us to more clearly emphasize the practical implications of our model. We agree that a patients’ molecular and cellular features such as endosomal volume, FcR expression in specific cells, etc. are not readily adjustable. Our intention was not to suggest therapeutics which modify these patient-specific features. Rather, we sought to identify sources of variability among patients that could be compensated for via personalized vaccine strategies. To clarify this point, we have significantly amended the analysis presented in Figure 6 to directly show how deficiency in FcRn or FcgRIIb expression can be compensated by changing tunable vaccine parameters such as timing, dosage, and adjuvant. We recognize that Fc receptor expression is not in and of itself adjustable; rather, such recommendations require future work to identify measurable biomarkers for these molecular regulators of transfer in easily accessible biospecimens such as maternal peripheral blood. Identification of such biomarkers is outside the scope of this study, but a natural future direction that we or others can pursue. In addition to substantially revising Figure 6, we have adjusted the verbiage in the Discussion section to make the directly or indirectly actionable clinical implications of this work clearer (see **lines 489-498**).

As the reviewer suggested, there are a limited number of parameters that can be tuned to the needs of specific patients. In regards to the “personalized medicine” approaches we allude to in the Introduction and Discussion sections, we envision personalized approaches which account for readily ascertainable patient-specific data, including: maternal vaccination/history of infection, baseline titer of antigen-specific antibodies and number of memory B cells, and risk and/or history of preterm delivery to inform when and how many vaccine doses should be administered. We have added sentences in both the Introduction and Discussion sections to clarify the feasibility and practical implementation of such personalized immunizations (see **lines 501-504**).

What is the rationale for only including FcgRIIb on ECs in the model? The authors have pointed out in Fig. 3 that the ECs express a mixture of FcRn and FcgRIIb, and the possible involvement of other FcgR (such as RIIIa). It seems strange that the FcRn/FcgRIIb model was only proposed in Fig. 3 but not the original model. Given these other possibilities are not ruled out as inconsistent with the data, it is not possible to determine whether the resulting model is the only one consistent with the evidence.

We appreciate the reviewer pointing out this inconsistency in our manuscript, which has prompted us to revise the model and amend our assumptions. The original rationale for including only FcgRIIb (not FcRn) in the ECs was that we discovered FcgRIIb expression to be a rate-determining parameter and thus decided to present the simplest model consistent with the data from Malek *et al*. However, we agree that the expression of FcRn is undeniable in ECs based on the scRNA-seq data presented in Fig 3 and that co-expression of FcRn with FcgRIIb is equally feasible. Although the mechanisms of receptor-mediated transcytosis in placental ECs are not yet fully characterized, evidence from literature suggests both FcRn and FcgRIIb are capable of IgG transcytosis in placental ECs independent of one other (3–5). Therefore, we revised the baseline model presented in Fig 1 to include both FcRn and FcgRIIb expressed by ECs, which are engaged in distinct mechanisms of transcytosis reflecting their ability to bind IgG at the cell surface (FcgRIIb) or intracellularly at an acidic pH (FcRn). The changes to the model are depicted in Fig 1A and in the equations contained in the supplemental appendix. Using this revised model structure, we performed a thorough parameter sweep using CaliPro and selected the parameter set with the highest percentage of model runs within the user-defined pass criteria (94%). This parameter set is presented in Table 1 of the revised manuscript. We used this new parameter set and revised model structure to update the analyses throughout the paper (Figs 1-3 and 5-6).

We would like to next address the Reviewer’s comment regarding the role of FcgRIIIa in STB transcytosis. While a few recent studies (including ours (6)) have provided statistical evidence which implicates a potential role for FcgRIIIa, these studies lack mechanistic insight. We do not find the purely associative data sufficient to justify the inclusion of FcgRIIIa in our mechanistic model. Although the role of FcgRIIb is similarly contested in the literature, a series of recent studies (3,5,7) characterize the role of FcgRIIb in EC transcytosis using a variety of microscopy and genetic engineering approaches.

We appreciate that the mechanisms of placental IgG transfer are not fully characterized to-date, hence the need for more studies driven by innovative computational and systems biology approaches in the future. This is the first piece of what we anticipate will be a collection of exciting work by us and others that leverages computational modeling to shed light on placental IgG transport. We have added a few sentences in the Discussion section to highlight opportunities for future studies to fill some of these knowledge gaps of how Fc receptors, including FcgRIIIa, dynamically regulate IgG transfer (see **lines 458-463**).

Observing a distinct optimum in vaccination timing seems very dependent on vaccination leading to a highly transient spike in antibodies which is, in turn, dependent on the proportion of short-lived ASCs. 96% was used in this study, but the cited study observes 68%–95%. Given the importance of this parameter, it should be discussed.

We appreciate the reviewer calling this to our attention and agree that the proportion of short-lived ASCs (ρ) has a substantial impact on the kinetics of the IgG response to vaccination, especially with regard to the transient spike in antibodies. The reviewer correctly identified that ρ is variable across patient cohorts, as is shown in White *et al* (8). However, it should also be noted that the models fit by White *et al* with different values of ρ also showed variation in

parameters including short- and long-lived ASC half-life and IgG half life, suggesting that this parameter is linked with other aspects of the IgG response.

Nevertheless, we agree it is important to address antibody responses characterized by different values of ρ and the broader impact of inter-patient variability in vaccine responses. To this end, we performed two separate *in silico* experiments to investigate the effect of ρ as well as peak antibody response on the optimal vaccination timing, the results of which are included as new supplemental figures S6 and S7, respectively:

- First, we adjusted the value of ρ by itself to the lower limit, mean, and upper limit of the range reported in White *et al.* We observed that the IgG response kinetics were sensitive to ρ. Low values of ρ resulted in a sustained increased level of anti-PT IgG in the maternal blood due to a higher percentage of long-lived ASCs. In this extreme case, the short, transient spike in maternal anti-PT IgG is essentially absent and we determined the optimal immunization time occurs at 10-12 weeks gestation. However, the kinetics of the anti-PT IgG response to Tdap vaccination observed by van der Lee *et al* are indeed characterized by a transient spike.
- Per the Reviewer's suggestion, we thought it prudent to simulate the extreme cases of the anti-PT IgG response magnitude observed in patients. To this end, we fit an IgG response curve to the lower and upper 95% confidence intervals of the Tdap cohort in van der Lee *et al*. This was possible by tuning only the k_IgG parameter (rate of IgG antibody secretion). As expected, changing only the magnitude of the peak affected the concentration of fetal anti-PT IgG at delivery, but did not impact the optimal immunization window.

Together, these *in silico* experiments demonstrate that vaccine recommendations depend upon the existence of a transient spike in IgG characterized by a high value of ρ, whereas the magnitude of this spike does not affect the optimal gestational immunization window. While anti-PT IgG responses to Tdap immunization were typically found to have a transient spike, other vaccines or responses against other antigens may follow distinct trends. We conclude that immunization recommendations should therefore be designed on a case-by-case basis. Fortunately, our modeling platform is modular, which enables any vaccine response and subsequent IgG transfer to be simulated given there is sufficient data to describe the maternal IgG kinetics. In addition to including results of these in silico explorations as new supplemental Figures S6 and S7, we have updated the verbiage throughout the Abstract, Results, and Discussion to clarify that our findings are specific to Tdap immunization and may not generalize to other vaccines (see **lines 15-16, 347-352,** and **470-477**).

The authors treat FcgRIIb and FcRn identically aside from their differing affinities at neutral pH. However, FcRn is highly pH-dependent, leading to its unique role in endosomal transport. Is there evidence to justify handling the endosomal trafficking for each of these receptors in the same way?

We appreciate the reviewer calling this discrepancy to our attention and acknowledge that FcRn and FcgRIIb are distinct in their cellular localization and mechanisms of binding to IgG. Respectfully, we would like to highlight that our original model captured this distinction in that FcRn expressed by STBs bound IgG within STB endosomes at an acidic pH, and the K_D value assigned to FcRn represents its affinity at low pH (9,10). This assumption was stated in the Methods section of the original draft. We presume the reviewer's comment is directed towards one of two flaws in our model, both of which are corrected in the revised manuscript:

- When we incorporated FcRn into the endothelial cells in Figure 3, we modeled its mechanism of transcytosis similar to that of FcgRIIb (binding at the surface followed by uptake and transcytosis). We have corrected this to ensure that the mechanism of FcRn-mediated transcytosis is consistent across STB and EC layers.
- While we assumed FcRn binding within endosomes and FcgRIIb binding at the cell surface, we incorrectly used the parameter k_up to describe both the rate of non-specific internalization of IgG prior to FcRn binding as well as the rate of internalization of FcgRIIb-IgG complexes. We adjusted our model equations such that k_up specifically describes that rate of non-specific IgG uptake, whereas FcR-IgG complexes transfer through the cell at a rate k_trans.

These distinct mechanisms of FcRn- and FcgRIIb-mediated transcytosis are substantiated by work by Ishikawa *et al* and Antohe *et al* who describe each pathway independently in placental endothelial cells. We acknowledge that while the mechanism of FcgRIIb-mediated transcytosis in placental endothelial cells has not yet been thoroughly characterized, the treatment of FcgRIIb in our model is consistent with its (i) role in endocytosis in liver sinusoidal endothelial cells, B cells, and macrophages (11), (ii) demonstrated colocalization with IgG in placental ECs (3), and (iii) ability to mediate transcytosis in HUVEC independent of FcRn . In our future work we plan to add to this growing body of literature by characterizing the dynamics of FcRn and FcgRIIb mediated transcytosis of IgG subclasses *in vitro*; we have made mention of this in the Discussion section (**lines 461-463**).

The schematic in Figure 1A has been adjusted to clearly delineate these pathways of FcRn- and FcgRIIb-mediated transcytosis. To accommodate this change to our model (as well as inclusion of both FcRn and FcgRIIb in EC transfer), we identified a new set of parameters which are presented in Table 1 (described in detail above). We revised the Results and Discussion sections accordingly and presented updated analyses using this improved model structure throughout the paper, most notably in Figures 1-3, 5, and 6.

Minor issues:

In Fig. 1A, is k_trans the same as k_t in the table? Keep the notation consistent.

Yes, k_trans and k_t are the same parameters. We apologize for the confusion, and have updated the notation in Table 1 to be consistent throughout the text.

In this same vein, we updated equations in Appendix S1 and added superscripts to a few variables for the sake of clarity. We changed Fig 1A to be consistent with these changes. We also added blocks to the schematic in Fig 1A to show generation of FcRn and FcgRIIb as described in Appendix S1, and distinguish between free and total (free+bound) FcRn/FcgRIIb.

Whether the model used in Fig. 4 for HUVEC is the same or different from the original one should be clearly stated.

These models are distinct. The placental model analyzed in Figures 1-3 and 5-6 is comprised of STBs and ECs together and is scaled to human pregnancy, whereas the model presented in Figure 4 recapitulates our *in vitro* model of IgG transcytosis in HUVEC and was parameterized by fitting to *in vitro* data. We have added a clarifying sentence in the Results section to delineate the model used in Fig. 4 from the original model, and a reference to the Methods where the full set of model equations used in Fig. 4 are listed (**line 236**). Along these lines, we made minor modifications to the model schematic in Figure 4 to improve interpretability.

In Fig. 5/Table 3, are decay rates d or δ? Keep the notation consistent.

Yes, decay rates d and δ are the same parameters. We apologize for the confusion and have updated notation in Figure 5 to be consistent with Table 3 (using δ to indicate decay rates).

In Table 1, K_D were listed as parameters, while the equations used k_on and k_off. How were the latter derived?

We thank the reviewer for the opportunity to rigorously justify our assumptions. Due to the very rapid kinetics of IgG-FcgR interactions, it is difficult to precisely ascertain k_on and k_off (12). Therefore, we made the simplifying assumption that all IgG-FcR interactions in our model have the same k_off value which has been reported for IgG1-FcRn (13). This is justified by evidence from other studies where one or more IgG subclasses were examined (14,15) or multiple species were compared (16,17). Using the value k_off = 0.149 (the mean of 3 mAbs studied in Suzuki *et al*) and the K_D values reported in Bruhns *et al*, we derived k_on using the equation k_on = k_off / K_D. To clarify this for the reader, we included this information in the Methods section and updated Table 1 to include k_on and k_off for each interaction along with relevant citations (**lines 566-570**).

Fig. 3 caption, line 211, did you mean "Statistical significance in (A-B)"?

Yes, we apologize for the confusion and have updated Fig. 3 caption accordingly.

Fig. S3 is an interesting result. Consider including it in Fig. 3.

We appreciate this suggestion and have included Fig S3 as part of panel C in Fig 3.

Fig. 4A, I wouldn't say the effect is "proportional" in line 221. It is hard to say there is a specific relation like this with just two examples.

We agree this wording is more precise and have changed the wording accordingly (now **lines 233-234**).

Is Fig. 4A the same as Fig. S4A except only showing IgG1 and IgG4? What is the composition of subclasses in "Competition"? Is the competition among two subclasses or four?

In our original draft, Fig. 4A is the same as Fig. S4A; both are simulations from the full placental compartmental model, as we had described in the first sentence of Fig. 4 caption. The composition of subclasses in "Competition" in both scenarios is among 4 subclasses. However, we realize that presenting the same model results in both Figure 4A and S4A may be a source of confusion. To simplify the data presentation, we omitted panel A from Fig 4 so that Fig 4 displays results only from our *in vitro* experiments and corresponding model representation of the *in vitro* system, whereas Fig S4A is *in silico* simulations used to motivate the *in vitro* work.

In Fig. 4D, it'd be helpful to mark where [IgG4_apical] > 0.33 is.

We appreciate this suggestion. Since the critical point occurs when [IgG_total] = [IgG1] + [IgG4] > 0.33, we decided to instead mark where [IgG1] = 0.13 with a dashed line, which corresponds to [IgG_total] = [IgG1] + [IgG4] = 0.13 + 0.2 = 0.33. After moving Fig. 4A to the Supplements, not Figure 4D is called Figure 4C.

Fig. 6C: would it make more sense to draw the thicker line (cyan or blue) on the x-axes?

In the revised version of Figure 6C, we decided that the information contained in the contour plots and the cross-sectional views was redundant, and the contour plots did not provide any added information towards our model-driven insights. As such, we provide only the line plots for the three cell type-specific receptors, which have changed from the previous submission due to the inclusion of the model with both FcγRIIb- and FcRn-mediated transfer in ECs.

In Fig. 1B, data were overlaid on the simulated levels. Don't see a description or a citation on the details of the data used.

This is correct, and we apologize for the mistake; we had referenced the source of the data (Malek *et al*, (29) in the original manuscript) (18) in the Results and Methods sections, but did not clearly explain that was the data included in Fig 1B. To clarify for the reader, we included a legend in Fig 1B that references the data source, added a citation in **line 104** in the Results section, and added a sentence in the Methods section specifically referencing the data set used for parameter estimation.

Did Fig. 3a and 3b use the same data? If so, why was "< 16 weeks" not included in Fig. 3a?

Fig 3A and Fig 3B use the same data modality (scRNA-seq). However, the data comes from two separate data sets and thus our ability to compare them directly is limited. The "<16 weeks" samples were obtained from Vento-Tormo *et al* and the "30 weeks" and ">37 weeks" samples from Tsang *et al* (19,20). In an effort to minimize bias introduced from batch effects, we felt it appropriate to only compare raw counts across samples obtained in the same study (Fig 3A). On the other hand, the relative expression of FCGR2B:FCGRT transcripts (Fig 3B) can reasonably be compared across studies. This was defined as the ratio of the expression of these two genes measured in each study.

In Fig. 3b, are "< 12 weeks" samples in line 174 the same as "< 16 weeks" in the figure? Are the "38 weeks" samples in line 176 the same as "> 37 weeks"?

Yes, and we apologize for this discrepancy. The labels in Fig. 3B ("<16 weeks", ">37 weeks") are accurate and we have changed the wording in **lines 188** and **190** to reflect this.

In lines 224-226, the rationale for choosing IgG1 and IgG4 should have citations.

We have added two references to support this rationale as indicated (now **line 239**).

For HUVEC in the Transwell assays, are the "three independent replicates" biological or technical?

Three biological replicates were assayed. We have updated the wording in Fig. 4 caption (**lines 276 and 281**) to clarify this.

Reviewer #2

In this manuscript, the authors have modelled transplacental antibody transfer to optimize immunization of newborns via vaccination of mothers. The authors have identified expression of

FcγRIIb on the endothelial cells to be a key limiting factor shaping the observed selectivity of different IgG subclasses. Combining this model with a vaccination model, the authors have identified an optimal gestational age range for vaccination of mothers to maximize neonatal immunity. Overall, I found this manuscript very well-written. Below are some minor comments, which should be addressed before publication.

Minor comments:

How can the values of FcRn and FcγRIIb expression be optimized as reported in Table 1, I thought they are variables in the model? According to the complete model equations in the SI, parameters a,b, and c needs to be estimated for the expression of FcRn and FcγRIIb.

We thank the reviewer for this comment because it allowed us to clarify our model structure for the reader. The values of FcRn and FcγRIIb reported in Table 1 are the optimized final (full-term) concentrations expressed on STBs and ECs. These values were determined using CaliPro similar to other parameters in the model, which is described in the Methods section. We would like to take this opportunity to clarify that FcRn and FcgRIIb are dynamic "offline" variables which increase by a second-order polynomial with time. For each simulation, FcRn or FcgRIIb at t=t_end is an input to the model. A second-order polynomial curve fit is used to determine parameters a, b, and c given constraints at t = 2 and 28 weeks. The polynomials are then evaluated at each time step in the model to yield different expression levels of FcRn and FcgRIIb as gestation progresses.

To make this clearer to the reader, we amended the Supplemental Information to describe the constraints used for curve fitting. We also provided more descriptive names for our parameters (e.g., $FcRn_{STB}^{total,end}$ rather than FcRn) to convey that the optimized parameters represent final concentrations only. In accordance with the description in the Supplementary Information (SI), we updated Fig 1A to visually convey that FcRn and FcgRIIb are newly synthesized and total expression increases (ΔFcRn and ΔFcgRIIb). Finally, we added a new supplemental figure (now Fig S1) to complement the description in SI and demonstrate our curve fitting process to obtain dynamic trends for FcRn and FcgRIIb.

In summary: Free unbound receptor is a variable, total receptor (bound+unbound) is an offline variable (provided as offline variable that changes as the placenta grows), and total receptor at birth is an estimated model parameter.

In table 1, for the parameters taken from published research, mainly from references 29 and 31, it will be good to know how these parameter values were calculated. If the values were directly taken from these references, please describe how they calculated the values. I suggest describing this in the Methods section.

We thank the reviewer for this comment which allows us to increase transparency of our model development. Per the reviewer's suggestion, we have added these descriptions in the Methods section. To address their question specifically about (29) and (31) (now (32) and (37)), we provide the following:

IgG concentrations used as initial conditions in the model were readily ascertainable from reference (29/32), where the authors directly measured IgG concentrations in maternal serum using ELISA. K_D values were readily ascertainable from reference (31/37) and were experimentally determined in vitro using SPR. While k_{off} and k_{on} values were not available for each IgG subclass-receptor combination in (31/37), we extracted k_{off} and k_{on} parameters

determined using SPR from published literature (Suzuki et al) under the simplifying assumption that all subclasses have the same k_off with FcRn and the same (but distinct) k_off with FcgRIIb; our assumptions and justification are described in more detail in response to a comment from Reviewer 1 above and have been incorporated into the Methods (**see lines 563-575**). Parameter δ_{AB} was derived from the half-life of IgG1 in infants, calculated in (32) (now (39)).

Please mention how many parameters were estimated and how many parameters were taken from literature in total.

In total, 11 parameters were optimized and 17 parameters were taken from literature. These are indicated in Tables 1-3 on the column called "Source" (specific optimized parameters slightly updated for this resubmission). Initial conditions are also listed in Table 1 and are directly obtained from (32).

In Fig 2, it is not clear how to interpret different points in fig A and C. Please explain briefly how OPLSR works.

We have added a brief subsection to the Methods to describe the use of Latin Hypercube Sampling (LHS) of the parameter space followed by OPLSR for the purpose of global sensitivity analysis, how to interpret the sensitivity analysis results, as well as references to publications which describe the method in greater detail. These changes can be found in **lines 587-614**. We also added an additional descriptive phrase both in the Results text as well as the Fig 2 caption to aid the reader.

References:

1. Eberhardt CS, Blanchard-Rohner G, Lemaître B, Boukrid M, Combescure C, Othenin-Girard V, et al. Maternal Immunization Earlier in Pregnancy Maximizes Antibody Transfer and Expected Infant Seropositivity Against Pertussis. Clin Infect Dis [Internet]. 2016 Apr 1 [cited 2022 Apr 6];62(7):829–36. Available from: https://academic.oup.com/cid/article/62/7/829/2463039
2. Stapleton NM, Andersen JT, Stemerding AM, Bjarnarson SP, Verheul RC, Gerritsen J, et al. Competition for FcRn-mediated transport gives rise to short half-life of human IgG3 and offers therapeutic potential. Nat Commun 2011 21 [Internet]. 2011 Dec 20 [cited 2022 Dec 9];2(1):1–9. Available from: https://www.nature.com/articles/ncomms1608
3. Ishikawa T, Takizawa T, Iwaki J, Mishima T, Ui-Tei K, Takeshita T, et al. Fc gamma receptor IIb participates in maternal IgG trafficking of human placental endothelial cells. Int J Mol Med [Internet]. 2015 May 1 [cited 2021 Jul 22];35(5):1273–89. Available from: http://www.spandidos-publications.com/10.3892/ijmm.2015.2141/abstract
4. Antohe F, Rădulescu L, Gafencu A, Ghe V, Simionescu M. Expression of functionally active FcRn and the differentiated bidirectional transport of IgG in human placental endothelial cells. Hum Immunol [Internet]. 2001 [cited 2022 Jun 7];62(2):93–105. Available from: https://pubmed.ncbi.nlm.nih.gov/11182218/
5. Takizawa T, Anderson CL, Robinson JM. A Novel FcγR-Defined, IgG-Containing Organelle in Placental Endothelium. J Immunol [Internet]. 2005 Aug 15 [cited 2021 Apr

29];175(4):2331–9. Available from: http://www.jimmunol.org/content/175/4/2331

6. Jennewein MF, Goldfarb I, Dolatshahi S, Cosgrove C, Noelette FJ, Krykbaeva M, et al. Fc Glycan-Mediated Regulation of Placental Antibody Transfer. Cell [Internet]. 2019;178(1):202-215.e14. Available from: https://doi.org/10.1016/j.cell.2019.05.044
7. Lyden TW, Robinson JM, Tridandapani S, Teillaud J-L, Garber SA, Osborne JM, et al. The Fc Receptor for IgG Expressed in the Villus Endothelium of Human Placenta Is FcγRIIb2. J Immunol [Internet]. 2001 Mar 15 [cited 2023 May 26];166(6):3882–9. Available from: https://journals.aai.org/jimmunol/article/166/6/3882/70424/The-Fc-Receptor-for-IgG-Expressed-in-the-Villus
8. White MT, Griffin JT, Akpogheneta O, Conway DJ, Koram KA, Riley EM, et al. Dynamics of the Antibody Response to Plasmodium falciparum Infection in African Children. J Infect Dis [Internet]. 2014 Oct 1 [cited 2022 Sep 22];210(7):1115–22. Available from: https://academic.oup.com/jid/article/210/7/1115/2908670
9. Vaccaro C, Bawdon R, Wanjie S, Ober RJ, Ward ES. Divergent activities of an engineered antibody in murine and human systems have implications for therapeutic antibodies [Internet]. 2006. Available from: https://www.pnas.org
10. Yang D, Giragossian C, Castellano S, Lasaro M, Xiao H, Saraf H, et al. Maximizing in vivo target clearance by design of pH-dependent target binding antibodies with altered affinity to FcRn. MAbs [Internet]. 2017;9(7):1105–17. Available from: https://doi.org/10.1080/19420862.2017.1359455
11. Mousavi SA, Sporstøl M, Fladeby C, Kjeken R, Barois N, Berg T. Receptor-mediated endocytosis of immune complexes in rat liver sinusoidal endothelial cells is mediated by FcγRIIb2. Hepatology. 2007 Sep;46(3):871–84.
12. Forest-Nault C, Gaudreault J, Henry O, Durocher Y, Crescenzo G De. On the Use of Surface Plasmon Resonance Biosensing to Understand IgG-FcγR Interactions. Int J Mol Sci 2021, Vol 22, Page 6616 [Internet]. 2021 Jun 21 [cited 2021 Oct 6];22(12):6616. Available from: https://www.mdpi.com/1422-0067/22/12/6616/htm
13. Suzuki T, Ishii-Watabe A, Tada M, Kobayashi T, Kanayasu-Toyoda T, Kawanishi T, et al. Importance of Neonatal FcR in Regulating the Serum Half-Life of Therapeutic Proteins Containing the Fc Domain of Human IgG1: A Comparative Study of the Affinity of Monoclonal Antibodies and Fc-Fusion Proteins to Human Neonatal FcR. J Immunol [Internet]. 2010 [cited 2023 Aug 29];184:1968–76. Available from: www.jimmunol.org/cgi/doi/10.4049/jimmunol.0903296
14. Zinkhan S, Thoms F, Augusto G, Vogel M, Bachmann MF. On the role of allergen-specific IgG subclasses for blocking human basophil activation. Front Immunol. 2022 Oct 6;13:892631.
15. Derebe MG, Nanjunda RK, Gilliland GL, Lacy ER, Chiu ML. Human IgG subclass cross-species reactivity to mouse and cynomolgus monkey Fcγ receptors. 2018 [cited 2023 Aug 29]; Available from: https://doi.org/10.1016/j.imlet.2018.02.006
16. Szikora B, Hiripi L, Bender B, Kacskovics I, Iliás A. Characterization of the interactions of rabbit neonatal Fc receptor (FcRn) with rabbit and human IgG isotypes. PLoS One [Internet]. 2017 Sep 1 [cited 2023 Aug 29];12(9):e0185662. Available from: https://journals.plos.org/plosone/article?id=10.1371/journal.pone.0185662
17. Datta-Mannan A, Witcher DR, Tang Y, Watkins J, Wroblewski VJ. Monoclonal Antibody Clearance IMPACT OF MODULATING THE INTERACTION OF IgG WITH THE NEONATAL Fc RECEPTOR *. 2007;

18. Malek A, Sager R, Kuhn P, Nicolaides KH, Schneider H. Evolution of maternofetal transport of immunoglobulins during human pregnancy. Am J Reprod Immunol. 1996;36(5):248–55.

19. Vento-Tormo R, Efremova M, Botting RA, Turco MY, Vento-Tormo M, Meyer KB, et al. Single-cell reconstruction of the early maternal–fetal interface in humans. Nature [Internet]. 2018 Nov 15 [cited 2021 Jan 15];563(7731):347–53. Available from: https://pubmed.ncbi.nlm.nih.gov/30429548/

20. Tsang JCH, Vong JSL, Ji L, Poon LCY, Jiang P, Lui KO, et al. Integrative single-cell and cell-free plasma RNA transcriptomics elucidates placental cellular dynamics. Proc Natl Acad Sci U S A [Internet]. 2017 Sep 12 [cited 2021 Mar 29];114(37):E7786–95. Available from: www.pnas.org/cgi/doi/10.1073/pnas.1710470114
